# Work impairment, osteoarthritis, and health-related quality of life among employees in Japan

**DOI:** 10.1186/s12955-018-0896-9

**Published:** 2018-04-17

**Authors:** Ken Nakata, Toshinaga Tsuji, Jeffrey Vietri, Dena H. Jaffe

**Affiliations:** 1 Medicine for Sports and Performing Arts, Osaka University Graduate School of Medicine, Suita, Japan; 2 Medical Affairs Department, Shionogi & Co., Ltd., Osaka, Japan; 3 Health Outcomes Practice, Kantar Health, Horsham, PA USA; 4 Health Outcomes Practice, Kantar Health, Ariel Sharon Street 4, 5230048 Tel Aviv, Israel

**Keywords:** Osteoarthritis, Presenteeism, Absenteeism, Health-related quality of life, Depression, Work impairment

## Abstract

**Background:**

Osteoarthritis (OA) is one of the most common causes of health and work impairment; however, this relationship, especially in Japan, is not well characterized. This study examined work impairment and OA in Japanese workers, specifically the relationship with health-related quality of life (HRQoL) and health status.

**Methods:**

This retrospective, cross-sectional observational study included the data of employed adults with a self-reported OA diagnosis from the 2014 Japan National Health and Wellness Survey. Presenteeism and absenteeism were classified using the Work Productivity and Activity Impairment (WPAI) questionnaire for impairment at work in the past week. Outcome variables included health-related quality of life, which was measured with the revised Medical Outcomes Study 36-Item Short Form Survey Instrument Health Survey (SF-36v2), and depression symptom severity, which was assessed using the Patient Health Questionnaire-9 (PHQ-9).

**Results:**

The majority (71.2%) of respondents with OA reported presenteeism, and 11.1% reported absenteeism. Presenteeism and absenteeism were both associated with younger age; a lower proportion of respondents with than without presenteeism were married or living with a partner, and a greater proportion of those with absenteeism had comorbid conditions (for all, *p* < 0.05). Respondents with than without presenteeism reported greater use of medications to relieve OA symptoms (37.3% versus 20.9%, *p* < 0.05), and those with than without absenteeism reported more frequent arthritis-related problems (*p* = 0.032). Among those with presenteeism, depression severity was higher (5.8 ± 6.0) than for those with no presenteeism (2.9 ± 4.3; *p* < 0.001). Presenteeism was associated with impairments in HRQoL on all metrics for patients with OA, with lower mental (6.4 points) and physical (4.8 points) component scores on the SF-36v2 (for all, *p* < 0.001).

**Conclusions:**

Seven out of every 10 patients with OA experienced presenteeism, whereas one out of 10 reported absenteeism. OA respondents with presenteeism also showed greater medication use, lower HRQoL across both mental and physical components, and higher depression severity. Workplace interventions and effective treatment options are necessary strategies for improving the health of workers with OA in Japan.

## Background

Osteoarthritis (OA) is a degenerative joint disorder that causes joint pain, stiffness, and restricted movement, and influences health-related quality of life (HRQoL) [1, 2]. According to a recent large-sample review of studies of patients with OA, impairment in HRQoL remains a significant component of the disease burden [3]. Moreover, OA comprises most of the economic loss that is due to arthritis, and by 2020, it is expected to be the world’s fourth main cause of disability [4]. It is anticipated that OA will have an even greater effect on health care systems and delivery of health care in the years beyond [2]. In Japan specifically, the prevalence of OA ranges from 10.0% to 62.4% in middle-aged and older individuals, however, OA is present in younger groups, as well [5–7].

Although OA is viewed as an age-related condition, there is a growing awareness that OA also affects younger individuals who are still employed [8]. Due to the impact of OA on employed individuals, the consideration of indirect costs stemming from productivity losses is an important aspect of assessing OA’s economic effects [9]. Indirect costs typically result from absenteeism and presenteeism [10]. Absenteeism represents time off from work caused by health-related non-attendance, disability, and/or workers’ compensation. Presenteeism assesses health-related productivity losses that occur while employees are at work [11].

A study examining the effect of OA on work productivity in five European countries reported about 33% of participants endorsing joint OA were employed [12]. Overall, arthritis-related pain is one of the most frequent pain-related conditions leading to impaired productivity among employed individuals [13]. Arthritis-related pain has been identified as a reason for leaving employment [14]. Furthermore, the pain and inhibited functioning caused by OA can result in lower worker productivity and early retirement [9], which can subsequently create significant economic losses for employers.

Studies in the US and Europe have reported that OA has a significant impact on worker absenteeism and presenteeism. Workers who were diagnosed with OA and experienced arthritis pain in the last month reported significantly more work impairment than those without OA pain, which was mainly due to presenteeism [8, 15, 16]. Similarly, prior research in five European countries reported that employees with OA experienced reduced work productivity caused by absenteeism (reported by 7%) and presenteeism (reported by 24%) [12].

The patterns of results reported in the international literature are generally aligned with the limited data from Japan. Notably, in a large international survey of adults with musculoskeletal symptoms (CUPID study), 5% of the 2280 Japanese workers sampled had reported sickness absences for pain complaints during the past year [17]. Presenteeism among Japanese workers with orthopaedic pain (low back, knee, or limb pain) was found to be most pronounced for physical job demands, ranging from 22.4% to 26.5% in the prior 2 weeks [18].

However, there is evidence that absenteeism may not be a useful an indicator of work productivity loss in Japan, relative to Western nations. For instance, among patients with low back pain, which is one of the most common musculoskeletal pains, the prevalence of absenteeism was three times lower in Japan than in the United Kingdom [17]. These authors suggested that in Japan, presenteeism may have more utility than absenteeism as an indicator of work productivity loss.

Beyond the economic burden attributed to OA, prior research has also consistently shown that OA is linked to poor HRQoL [8, 15]. In a retrospective observational study, US patients with self-reported OA pain reported worse HRQoL, as demonstrated by significantly lower scores on the Physical Component Summary (PCS; 40.1 vs. 50.8), Mental Component Summary (MCS; 46.3 vs. 47.4), and Short Form-6 Dimension (SF-6D; 0.66 vs. 0.75), compared with controls [8]. Similarly, US adults with moderate or severe self-reported OA had PCS and SF-6D scores that were significantly lower than those of controls, signifying worse HRQoL [15].

Among Japanese adults, musculoskeletal pain has likewise been associated with decrements in HRQoL [19, 20]. For example, patients diagnosed with knee OA via Kellgren-Lawrence grades ≥3 and experiencing knee pain reported lower scores on the PCS and the EuroQoL, with higher scores on the Western Ontario and McMaster Universities Osteoarthritis Index, than patients with Kellgren-Lawrence grades ≤1 [20]. Additionally, among Japanese adults with a self-reported diagnosis of chronic lower back pain, those with severe pain had lower MCS, PCS, and SF-6D scores than counterparts with mild or moderate pain [19].

Several studies have examined the association between OA and HRQoL in Japan, and it has been well-established that OA is associated with a substantial health and economic burden. However, the relationship between work productivity loss (i.e., absenteeism and presenteeism) and HRQoL in this patient population is not well understood. Furthermore, despite the association between OA symptoms and lower work productivity, particularly presenteeism, a limited number of studies have reported on individual-level or work factors associated with presenteeism among those who have conditions like OA [21], especially in Japan. Instead, the presenteeism literature on Japanese populations has focused on other conditions or health-related factors, such as lower back pain [22, 23], depression [24, 25], outpatient chemotherapy treatment [26], and dry eyes [27]. This study aimed to address this gap by comparing those with OA in Japan who did versus did not report presenteeism across individual characteristics. Overall, the current study focused on the association between work productivity and HRQoL in employed Japanese patients with OA, which is one of the most prevalent musculoskeletal pains. Informed by the prior literature, we hypothesized that, among patients with OA, those with higher work productivity loss are likely to have poorer HRQoL, and the association between work productivity loss and HRQoL is stronger for presenteeism than for absenteeism.

## Methods

### Data source and sample

Data were obtained from the 2014 Japan National Health and Wellness Survey (NHWS, Kantar Health, New York, NY, USA), a large web-based cross-sectional online survey of individuals age 18 and older. Potential respondents to the NHWS are recruited through an existing web-based consumer panel. The consumer panel recruits its members through opt-in emails, co-registration with panel partners, e-newsletter campaigns, banner placements, and both internal and external affiliate networks. Participation is voluntary, and the NHWS uses stratified random sampling with quotas based on gender and age group to ensure the sample reflects the Japanese population, as reported in the US Census International Database. The NHWS includes epidemiological data, as well as data on socio-demographics, current and past medical history, treatment information, health risk behaviors, and health-related outcomes. The Japan NHWS in 2014 was reviewed and found exempt by Pearl IRB.

All responses to NHWS measures were self-reported. Of the total sample (*N* = 30,000), 565 respondents reported an OA diagnosis, and the final study sample included 233 respondents who reported receiving an OA diagnosis from a healthcare provider and were currently employed. No exclusion criteria were applied.

### Measures

#### Work productivity and activity impairment

Work productivity impairment was assessed using the Work Productivity and Activity Impairment (WPAI) questionnaire, a six-item validated instrument that consists of four metrics: presenteeism (the percentage of impairment experienced while at work in the past 7 d because of one’s health), absenteeism (the percentage of work time missed because of one’s health in the past 7 d), overall work productivity loss (an overall impairment estimate that is a combination of absenteeism and presenteeism), and activity impairment (the percentage of impairment in daily activities because of one’s health in the past 7 d) [28]. In addition to the continuous measure, presenteeism and absenteeism were categorized according to any versus no impairment for each variable.

#### HRQoL and health status

HRQoL was measured using the revised Medical Outcomes Study 36-Item Short Form Survey Instrument (SF-36v2) [29]. This is a multipurpose, generic HRQoL instrument comprised of 36 questions with summary scores. The present analysis included the standard, US-based summary scores for the MCS and PCS, which each have a mean of 50 and standard deviation of 10 in the US population. Scores can be interpreted relative to this population average of 50, as well as with other comparison groups of interest. Higher scores indicate better HRQoL. In addition, the instrument was also used to generate health state utilities, namely the SF-6D. This preference-based single index measure of health uses general population values; the current study used values based on the general population of the United Kingdom [30]. The SF-6D index has interval scoring properties and yields summary scores on a theoretical 0–1 scale (with an empirical floor of 0.3). Higher scores indicate better HRQoL. Finally, the SF-36v2 instrument is designed to report on eight health concepts (physical functioning, role physical, bodily pain, general health, vitality, social functioning, emotional role limitations, and mental health). The eight-factor health profile presented in the current study is based on the Japanese scoring norms, with a mean of 50 and standard deviation of 10 in the Japanese population; higher scores indicate better functional health [31].

Depression symptoms and severity of depression over the last 2 weeks were assessed using the Patient Health Questionnaire-9 (PHQ-9), a validated scale used to screen for depression and assess its severity [32]. This scale measures depression symptom severity through the frequency in the past 2 weeks of anhedonia, depressed mood, sleep disturbance, lack of energy, appetite disturbance, negative self-feelings, difficulty concentrating, psychomotor retardation or agitation, and thoughts of self-harm. Scores on the PHQ-9 can range from 0 to 27; higher scores indicate more severe depression symptoms.

#### Respondent characteristics

Age (in years), gender, marital status (married or living with partner vs. single, divorced, separated or widowed), annual household income (< ¥3million, ¥3million to < ¥5million, ¥5million to < ¥8million, ¥8million or more, or decline to answer), and level of education categorized at approximately the median (junior/high school, two-year college, four-year college, or graduate school)) were assessed for all respondents.

Body mass index (BMI) was calculated in kilograms per meter squared from reported height and weight and reported as underweight (< 18.5 kg/m^2^), normal weight (18.5 to < 25.0 kg/m^2^), overweight (25.0 to < 30.0 kg/m^2^), obese (30.0 kg/m^2^ and above), or decline to answer. Information for cigarette smoking was collected using the following two questions “Have you ever smoked cigarettes?” and “Do you currently smoke cigarettes?” These answers were then recoded into a smoking variable reflecting current, former, or never smokers. Alcohol use was based on the question “How often do you drink alcohol?” Responses options ranged from daily to once a month or less often; there was also an option of “I do not drink alcohol.” The alcohol use variable was recoded into none vs. any. Vigorous exercise at least 1 d in the past month was based on the question “How many days in the past month did you exercise vigorously for at least 20 minutes for the purpose of improving or maintaining your health, with the purpose of losing weight, or for enjoyment?” This was then recoded into a yes or no variable.

The Charlson Comorbidity Index (CCI) [33] weights the presence of the following conditions and sums the result: HIV/AIDS, metastatic tumor, lymphoma, leukemia, any tumor, moderate/severe renal disease, hemiplegia, diabetes, mild liver disease, ulcer disease, connective tissue disease, chronic pulmonary disease, dementia, cerebrovascular disease, peripheral vascular disease, myocardial infarction, congestive heart failure, and diabetes with end organ damage. The greater the total index score, the greater the comorbid burden on the patient.

OA-related characteristics included self-reported length of diagnosis in years, type (multiple sites could be provided) and number of joints affected, severity of arthritis (mild, moderate, or severe), frequency of problems with arthritis (daily, 4–6 times a week, 2–3 times a week, once a week, 2–3 times a month, or once a month or less often), and use of prescription medication for arthritis.

#### Analysis

All analyses were conducted using IBM Statistical Package for the Social Sciences (SPSS) version 22. Respondent sociodemographic and OA characteristics were described for the sample cohort using means and standard deviations for continuous and count variables, and frequencies and percentages for categorical variables. Respondents with and without presenteeism and those with and without absenteeism were compared on socio-demographic and health-related characteristics using t-tests for continuous variables and chi-square tests or Fisher exact tests (for cells with *n* < 5) for categorical variables. Cohen’s d was calculated to examine the magnitude of difference between groups; it is calculated as the difference in means divided by the standard deviations of each group [34]. A difference of 0.2 is considered small, with more expected overlapping values (92%) between comparison groups, while 0.8 is large, with less overlapping values (69%) between groups.

Regression analyses, which incorporated variables in the bivariate comparisons that were at least marginally significant at *p* < 0.10, were used to examine adjusted least-square means for HRQoL by level of presenteeism. Due to the relatively small comparison sample for those with and without absenteeism, further examination of health outcomes was restricted to those with and without presenteeism. The ordinary least square linear regression models analyzed health outcomes as a function of presenteeism (reference = no presenteeism vs. any presenteeism). These models were used to demonstrate the burden of presenteeism on patients with physician-diagnosed OA, controlling for confoundersfound to differ between groups at *p* < 0.1 in the bivariate analysis. This model was used, since HRQoL was normally distributed. Model fit was assessed according to a significant F-test. All analyses assumed a null hypothesis with a two-sided α < 0.05.

Correlations were calculated between presenteeism and other outcomes. Spearman’s rho was used because of the non-normal distribution of presenteeism.

## Results

A total of 233 employed, adult respondents with OA were examined, with 166 of 233 (71.2%) reporting presenteeism and 25 of 225 (11.1%) reporting absenteeism in the past week (Tables 1 and 2). Presenteeism and absenteeism were associated with younger age (presenteeism: any = 52.9 ± 12.4 years vs. none = 57.3 ± 11.2 years, *p* = 0.012; absenteeism: any = 46.1 ± 15.3 years vs. none = 55.4 ± 11.3 years, *p* < 0.001). The only other socio-demographic or health-related characteristics that differed between presenteeism groups were marital status and education. Specifically, a lower proportion of individuals with presenteeism was married or living with a partner (any = 62.7% vs. none = 76.1%, *p* = 0.049), and they had greater educational attainment (*p* = 0.017). Those with absenteeism reported a higher comorbidity burden (CCI scores) than OA patient with no absenteeism (any = 3.1 ± 8.0 vs. none = 0.7 ± 3.3; *p* = 0.006).

**Table 1:** Sociodemographic and osteoarthritis characteristics by presenteeism among respondents with osteoarthritis in Japan, 2014

			Presenteeism				
	Total (*N* = 233)		Presenteeism (*N* = 166)		No Presenteeism(*N* = 67)		*p*-value^*^
Female (%, N)	43.8%	102	46.4%	77	37.3%	25	0.206
Age (Mean, SD)	54.2	12.2	52.9	12.4	57.3	11.2	0.012
Married or living with partner (%, N)	66.5%	155	62.7%	104	76.1%	51	0.049
Annual household income (%, N)							0.987
< ¥3million	14.2%	33	13.9%	23	14.9%	10	
¥3million to <¥5million	20.6%	48	20.5%	34	20.9%	14	
¥5million to <¥9million	36.5%	85	37.3%	62	34.3%	23	
¥9million or more	21.0%	49	21.1%	35	20.9%	14	
Decline to answer	7.7%	18	7.2%	12	9.0%	6	
Education (%, N)							0.017
Junior/High School	30.5%	71	26.5%	44	40.3%	27	
2-year college	14.6%	34	16.3%	27	10.4%	7	
4-year college	49.4%	115	53.6%	89	38.8%	26	
Graduate school	5.6%	13	3.6%	6	10.4%	7	
CCI (Mean, SD)	1.1	4.8	1.0	4.4	1.5	5.6	0.462
BMI categories (%, N)							0.665
Underweight (< 18.5)	7.7%	18	7.2%	12	9.0%	6	
Normal (18.5–24.9)	61.4%	143	59.0%	98	67.2%	45	
Overweight (25.0–29.9)	22.7%	53	24.7%	41	17.9%	12	
Obese (30.0+)	6.9%	16	7.2%	12	6.0%	4	
Decline to answer	1.3%	3	1.8%	3	0.0%	0	
Smoking status (%, N)							0.518
Current smoker	26.2%	61	24.1%	40	31.3%	21	
Former smoker	34.3%	80	35.5%	59	31.3%	21	
Never smoker	39.5%	92	40.4%	67	37.3%	25	
Drinks alcohol (%, N)	75.5%	176	74.1%	123	79.1%	53	0.421
Exercise (%, N)	51.1%	119	51.8%	86	49.3%	33	0.724
Length of arthritis diagnosis, years^a^ (Mean, SD)	8.4	11.3	8.4	11.0	8.2	12.0	0.897
Number of joints affected (Mean, SD)	2.0	1.9	1.9	1.7	2.1	2.2	0.482
Site affected by arthritis (%, N)							
Ankles	15.9%	37	17.5%	29	11.9%	8	0.330
Elbows	13.3%	31	12.0%	20	16.4%	11	0.397
Feet	14.6%	34	14.5%	24	14.9%	10	1.000
Fingers/Hands	26.6%	62	25.3%	42	29.9%	20	0.477
Hips	14.2%	33	15.1%	25	11.9%	8	0.679
Knees	51.9%	121	50.6%	84	55.2%	37	0.564
Neck	10.3%	24	10.2%	17	10.4%	7	1.000
Shoulders	14.2%	33	12.0%	20	19.4%	13	0.151
Spine	3.0%	7	3.0%	5	3.0%	2	1.000
Wrists	16.7%	39	17.5%	29	14.9%	10	0.702
Other	6.0%	14	4.2%	7	10.4%	7	0.123
Severity of arthritis (%, N)							
Mild	51.5%	120	51.2%	85	52.2%	35	0.959
Moderate	38.6%	90	39.2%	65	37.3%	25	
Severe	9.9%	23	9.6%	16	10.4%	7	
Frequency of problems with arthritis (%, N)							0.520
Daily	33.0%	77	35.5%	59	26.9%	18	
4–6 times a week	7.7%	18	8.4%	14	6.0%	4	
2–3 times a week	12.9%	30	13.3%	22	11.9%	8	
Once a week	2.6%	6	3.0%	5	1.5%	1	
2–3 times a month	14.2%	33	12.0%	20	19.4%	13	
Once a month or less often	29.6%	69	27.7%	46	34.3%	23	
Use a prescription for OA (%, N)	32.6%	76	37.3%	62	20.9%	14	0.015

Note: *BMI* body mass index, *CCI* Charlson Comorbidity Index, *OA* osteoarthritis

*Differences between none vs. any presenteeism were performed using t-tests for continuous variables and chi-square or Fisher exact (cells < 5) tests for categorical variables

^a^Data for length of diagnosis were missing for an additional 12 respondents overall

**Table 2:** Sociodemographic and osteoarthritis characteristics by absenteeism among respondents with osteoarthritis in Japan, 2014

			Absenteeism^a^				
	Total (*N* = 233)		Absenteeism (*N* = 25)		No Absenteeism (*N* = 200)		*p*-value^*^
Female (%, N)	43.8%	102	52.0%	13	43.5%	87	0.420
Age (Mean, SD)	54.2	12.2	46.1	15.3	55.4	11.3	< 0.001
Married or living with partner (%, N)	66.5%	155	64.0%	16	68.0%	136	0.687
Annual household income (%, N)							0.204
< ¥3million	14.2%	33	8.0%	2	15.0%	30	
¥3million to <¥5million	20.6%	48	16.0%	4	21.0%	42	
¥5million to <¥9million	36.5%	85	32.0%	8	37.5%	75	
¥9million or more	21.0%	49	40.0%	10	18.0%	36	
Decline to answer	7.7%	18	4.0%	1	8.5%	17	
Education (%, N)							0.584
Junior/High School	31.1%	70	24.0%	6	32.0%	64	
2-year college	14.2%	32	20.0%	5	13.5%	27	
4-year college	50.2%	113	48.0%	12	50.5%	101	
Graduate school	4.4%	10	8.0%	2	4.0%	8	
CCI (Mean, SD)	1.1	4.8	3.1	8.0	0.7	3.3	0.006
BMI categories (%, N)							0.557
Underweight (< 18.5)	7.7%	18	8.0%	2	8.0%	16	
Normal (18.5–24.9)	61.4%	143	68.0%	17	60.5%	121	
Overweight (25.0–29.9)	22.7%	53	12.0%	3	23.5%	47	
Obese (30.0+)	6.9%	16	12.0%	3	6.5%	13	
Decline to answer	1.3%	3	0.0%	0	1.5%	3	
Smoking status (%, N)							0.055
Current smoker	26.2%	61	44.0%	11	23.0%	46	
Former smoker	34.3%	80	20.0%	5	37.0%	74	
Never smoker	39.5%	92	36.0%	9	40.0%	80	
Drinks alcohol (%, N)	75.5%	176	84.0%	21	75.0%	150	0.421
Exercise (%, N)	51.1%	119	56.0%	14	49.0%	98	0.509
Length of arthritis diagnosis, years^a^ (Mean, SD)	8.4	11.3	12.0	16.2	7.9	10.5	0.100
Number of joints affected (Mean, SD)	2.0	1.9	1.8	2.2	1.9	1.7	0.712
Site affected by arthritis							
Ankles	15.9%	37	20.0%	5	14.5%	29	0.551
Elbows	13.3%	31	12.0%	3	13.5%	27	1.000
Feet	14.6%	34	20.0%	5	14.0%	28	0.382
Fingers/Hands	26.2%	59	20.0%	5	27.0%	54	0.453
Hips	14.2%	33	24.0%	6	12.5%	25	0.126
Knees	51.9%	121	32.0%	8	54.0%	108	0.055
Neck	10.3%	24	8.0%	2	10.5%	21	1.000
Shoulders	14.2%	33	8.0%	2	15.0%	30	0.544
Spine	3.0%	7	4.0%	1	2.5%	5	0.511
Wrists	16.7%	39	20.0%	5	16.5%	33	0.584
Other	6.0%	14	8.0%	2	5.5%	11	0.643
Severity of arthritis (%, N)							0.356
Mild	51.5%	120	56.0%	14	52.0%	104	
Moderate	38.6%	90	28.0%	7	39.0%	78	
Severe	9.9%	23	16.0%	4	9.0%	18	
Frequency of problems with arthritis (%, N)							0.032
Daily	33.0%	77	24.0%	6	33.0%	66	
4–6 times a week	7.7%	18	16.0%	4	6.5%	13	
2–3 times a week	12.9%	30	32.0%	8	11.0%	22	
Once a week	2.6%	6	0.0%	0	3.0%	6	
2–3 times a month	14.2%	33	12.0%	3	15.0%	30	
Once a month or less often	29.6%	69	16.0%	4	31.5%	63	
Use a prescription for OA (%, N)	32.6%	76	44.0%	11	31.0%	62	0.015

Note: *BMI* body mass index, *CCI* Charlson Comorbidity Index, *OA* osteoarthritis

*Differences between none vs. any absenteeism were performed using t-tests for continuous variables and chi-square or Fisher exact (cells < 5) tests for categorical variables

^a^Data for absenteeism were missing for eight respondents. Data for length of diagnosis were missing for an additional 12 respondents overall

Most OA-related characteristics were similar between those with and without presenteeism or absenteeism (Tables 1 and 2). The use of prescription medication was substantially higher for those with presenteeism (37.3%) than those without (20.9%, *p* = 0.015), and OA-related problems were more frequent for those with absenteeism (*p* = 0.032). No statistically significant differences in sites affected by OA were observed for respondents with or without presenteeism or absenteeism.

Presenteeism was associated with impairments in HRQoL for all metrics at *p* < 0.001 for patients with OA (Table 3). Additionally, OA patients with presenteeism reported lower MCS (6.4 points) and PCS (4.8 points) scores than those with no presenteeism. In the overall SF-6D health utility index, patients with presenteeism had a 0.07-point lower score than those without presenteeism. Health status, according to the eight health domains, was substantially lower by 6.1–9.0 points for those with than without presenteeism. Those with presenteeism (5.8 ± 6.0) had greater depression symptom severity, as measured using the PHQ-9 than those without presenteeism (2.9 ± 4.3, *p* < 0.001).

**Table 3:** Health-related quality of life and depression by presenteeism among respondents with osteoarthritis in Japan, 2014

	Total (*N* = 233)		Presenteeism (*N* = 166)		No presenteeism (*N* = 67)			
	Mean	SD	Mean	SD	Mean	SD	*p*-value	Cohen’s d
MCS	46.2	10.6	44.4	11.2	50.8	7.4	< 0.001	0.67
PCS	47.5	7.5	46.1	7.8	50.9	5.5	< 0.001	0.71
Physical functioning	44.3	15.5	42.0	16.7	50.1	10.2	< 0.001	0.59
Physical role limitations	43.9	13.4	41.3	13.9	50.3	9.6	< 0.001	0.75
Bodily pain	42.1	10.1	40.2	9.7	46.7	9.6	< 0.001	0.67
General health	42.5	10.5	40.6	10.3	47.0	9.5	< 0.001	0.65
Vitality	44.2	10.9	42.4	10.8	48.6	10.0	< 0.001	0.46
Social functioning	44.8	12.9	42.5	13.4	50.7	9.3	< 0.001	0.71
Emotional role limitations	45.9	12.6	44.2	13.2	50.3	9.4	< 0.001	0.53
Mental health	46.2	11.4	44.1	11.6	51.3	8.8	< 0.001	0.70
SF-6D (health utility)	0.69	0.12	0.67	0.12	0.74	0.10	< 0.001	0.63
PHQ-9 total score (depression)	5.0	5.7	5.8	6.0	2.9	4.3	< 0.001	0.56

Cohen’s d reflects the difference between the means divided by the standard deviations for those with and without presenteeism. A difference of 0.2 is considered small, 0.5 is medium, and 0.8 is large. As Cohen’s d value increases, the lower percentage of overlapping values are observed between groups

Note: *MCS* Mental Component Summary, *PCS* Physical Component Summary, *PHQ-9* Patient Health Questionnaire-9, *SF-6D* Short-Form Six-Dimension

**Fig. 1:**
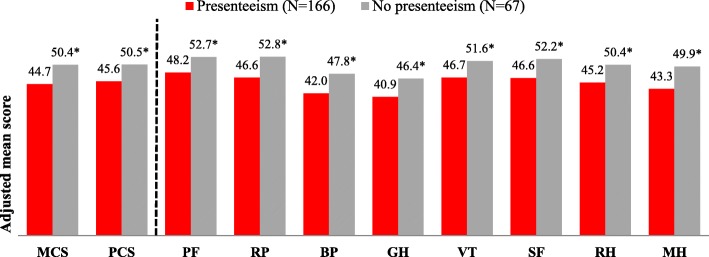
Adjusted means of health-related quality of life metrics for OA patients with and without presenteeism^a^. Note: MCS = Mental Component Summary; PCS=Physical Component Summary; PF = physical functioning; RP = physical role limitation; BP = body pain; GH = general health; VT = vitality; SF = social functioning; RH = emotional role functioning; MH = mental health. Generalized linear models using a normal distribution were adjusted for age, education, and marital status (married or living with a partner vs. other). ^a^Means were adjusted for age, education, and marital status. *Comparisons between groups were significant at *p* ≤ 0.001

Similar differences in HRQoL were observed in regression analyses after adjusting for covariates age, marital status, and education (Fig. 1). Adjusted means were lower for both the MCS and PCS, as well as for all eight measures of health status (*p* ≤ 0.001). Adjusted means for the SF-6D health utility score remained lower for those with (vs. without) presenteeism (0.67 ± 0.01 vs. 0.74 ± 0.02, *p* < 0.001).

Work and activity impairment, which were examined in relation to presenteeism, were substantially higher among employed OA respondents with, compared to without, presenteeism (Table 4). More patients with than without presenteeism reported absenteeism (15.1% vs. 1.5%, *p* = 0.001). Specifically, absenteeism and overall work impairment were greater among those with than without presenteeism (absenteeism: 2.9% ± 10.8% vs. 0.0% ± 0.4%, *p* = 0.034; overall work impairment: 39.5% ± 25.1% vs. 0.0% ± 0.4%, *p* < 0.001). Activity impairment was significantly higher among those with presenteeism than without (38.7% ± 25.3% vs. 6.7% ± 18.5%, *p* < 0.001).

**Table 4:** Work and activity impairment by presenteeism among respondents with osteoarthritis in Japan, 2014

	Total (*N* = 233)	Presenteeism (*N* = 166)	No presenteeism (*N* = 67)	*p*-value
Absenteeism^a^ (any), (n, %)	25 (11.1%)	24 (15.1%)	1 (1.5%)	0.002
Absenteeism^a^ (%), (mean ± SD)	2.1 ± 9.2	2.9 ± 10.8	0.0 ± 0.4	0.034
Presenteeism (any), (n, %)	166 (71.2%)	166 (100%)	0 (0%)	NA
Presenteeism (%), (mean ± SD)	27.0 ± 26.7	38.0 ± 24.3	0.0 ± 0.0	NA
Overall work impairment (%), (mean ± SD)	27.9 ± 27.7	39.5 ± 25.1	0.0 ± 0.4	< 0.001
Activity impairment (%), (mean ± SD)	29.5 ± 27.6	38.7 ± 25.3	6.7 ± 18.5	< 0.001

^a^There were eight respondents with missing data

When considered as continuous variables, presenteeism was negatively associated with all of the HRQoL indicators and positively associated with depression characteristics (for all, *p* < 0.001) (Table 5). Higher presenteeism was moderately associated with lower MCS, PCS, and SF-6D scores, with the strongest correlation being with health utility scores (rho = − 0.51, *p* < 0.001). Higher presenteeism was associated with lower functional health status on all eight SF-36v2 health profile scores using the Japanese norm-based scoring. These correlations ranged from rho = − 0.37 for emotional role limitations to rho = − 0.49 for physical role limitations, with other measures falling between these two extremes. Higher presenteeism was also moderately related to greater depression severity, based on PHQ-9 scores (rho = 0.42, *p* < 0.001).

**Table 5:** Correlations between presenteeism and health-related quality of life among respondents with osteoarthritis in Japan, 2014

	Correlation (r_s_)	*p*-value
MCS	−0.44	< 0.001
PCS	−0.46	< 0.001
SF-6D (health utility)	−0.51	< 0.001
Physical functioning	−0.38	< 0.001
Physical role limitations	−0.49	< 0.001
Bodily pain	−0.44	< 0.001
General health	−0.47	< 0.001
Vitality	−0.43	< 0.001
Social functioning	−0.48	< 0.001
Emotional role limitations	−0.37	< 0.001
Mental health	−0.45	< 0.001
PHQ-9 total score (depression)	0.42	< 0.001

Note: *MCS* Mental Component Summary, *PCS* Physical Component Summary, *PHQ-9* Patient Health Questionnaire-9, *SF-6D* Short-Form Six-Dimension

## Discussion

In the current study, 71.2% respondents with OA reported some degree of impairment at work, while only 11.1% reported missing work due to a health-related problem. The results show that, among employed adults, those with OA and presenteeism tend to be younger, not married or living with a partner, and report a greater use of OA medication. Respondents with presenteeism had substantially lower HRQoL than those without presenteeism.

This and other studies show that, while OA affects health outcomes, the larger impact to work impairment is from presenteeism and not absenteeism [8, 12, 15]. A study of Japanese workers with musculoskeletal pain hypothesized that presenteeism among workers in Japan may be more prevalent, compared with workers in other countries, due to cultural differences surrounding concerns about absence from work [16]. Thus, although respondents with absenteeism appear to have a greater health burden (i.e., higher CCI scores and more OA-related problems), few patients with OA report absenteeism. In contrast, presenteeism represents a larger proportion of OA patients, and their HRQoL burden is relatively high.

Overall, findings indicated that presenteeism is much more common than absenteeism among employed adults with OA in Japan. It is possible this may be due to those patients with less severe pain opting to remain at work, rather than to take time off to manage their OA pain. Nevertheless, this situation is problematic, as their productivity may be considerably reduced while on the job. Taken together, physicians should be sensitive to those patients with less severe OA who remain at work, but may suffer from increased presenteeism and reduced HRQoL. To this end, results suggest that presenteeism may serve as a useful indicator for physicians to identify those workers with OA who may require additional pain management.

The current study also revealed significant differences in current use of prescription medication for OA, with the presenteeism group using prescription medications (to alleviate OA symptoms) at a significantly higher rate than the non-presenteeism group. This result is consistent with other research that reported an association between work impairment and OA severity [35] and between daily functioning and pain levels among patients with OA [36]. Higher use of prescription drugs among the presenteeism group could be related to the greater pain intensity experienced by that group. Kingsbury et al. indicated that the use of prescription medication rises when the severity of pain increases [12].

Furthermore, our study indicates that the relationship between presenteeism and HRQoL may extend beyond the workplace. In addition to greater use of OA medications, the presenteeism group also had greater impairment to both physical and mental HRQoL, as well as higher depression scores, compared with the no presenteeism group. Taken as a whole, these data are consistent with models relating pain severity to behavioral, cognitive, and emotional sequelae and to pain management interventions that use these models [37]. To illustrate, individuals with higher levels of pain may experience difficulty sleeping, thereby decreasing concentration and increasing presenteeism at work. This result is also in line with research in Japan that detected an association between presenteeism and depression [25].

### Limitations

The data were collected using self-report and thus could be affected by recall bias. Inclusion criteria were based on patients’ self-reported physician diagnosis and employment status in order to minimize selection bias and reflect the ‘healthier’ OA patient; those who indicated ever experiencing OA, but without a self-reported diagnosis were not included in this study. While the study design likely represents healthier patients who are able to fill out a survey, the low percentage of patients with absenteeism is representative of OA in Japan and of the Japanese population [8, 12, 15]. The cross-sectional design of this study did not allow for causality to be assessed. Since the data were also collected using an internet-based survey, the sample may also not be fully representative of the population of interest. Moreover, as with any group of instruments that measure the same construct, variations are shown across measures of presenteeism [38]. It is thus a possibility that slightly different results may be shown if another presenteeism instrument were used [38]. The nature of a self-reported study may also under- or overestimate the severity of the disease, however it has been shown that for OA, patient-reported severity is an acceptable measure of perceived health and should be used by providers during care management [39]. As this was a retrospective study, analyses could only adjust for those confounding variables collected in the survey. Finally, sites of OA were collected. However, since respondents could report multiple OA-affected sites, and the overall sample was relatively small, we were unable to draw conclusions based on an individual site of arthritis pain.

## Conclusions

In this study, seven of every 10 OA patients experienced presenteeism, while only one out of 10 reported absenteeism. Presenteeism was greater among younger patients and those not married and not living with a partner. OA patients with than without presenteeism showed greater medication use, lower HRQoL across both mental and physical components, and higher depression scores. These findings demonstrate a need for workplace interventions and effective treatment options for workers in Japan who are diagnosed with OA.

## Abbreviations

BMI: Body mass index
CCI: Charlson Comorbidity Index
MCS: Mental Component Summary
NHWS: National Health and Wellness Survey
OA: Osteoarthritis
PCS: Physical Component Summary
PHQ-9: Patient Health Questionnaire-9
SF-36v2: Revised Medical Outcomes Study 36-Item Short Form Health Survey
SF-6D: Short-Form Six-Dimensions
SPSS: Statistical Package for the Social Sciences
WPAI: Work Productivity and Activity Impairment Questionnaire
